# Characterization changes and research waste in randomized controlled trials of global gastroesophageal reflux disease and hiatus hernia over the past 20 years

**DOI:** 10.1097/JS9.0000000000002227

**Published:** 2025-01-24

**Authors:** Bin Lin, Xiao-Jing Guo, Yi-Ming Jiang, Zhi-Xin Shang-Guan, Qing Zhong, Qi-Yue Chen, Jian-Wei Xie, Ping Li, Chao-Hui Zheng, Chang-Ming Huang, Jian-Xian Lin

**Affiliations:** a Department of Gastric Surgery, Fujian Medical University Union Hospital, Fuzhou, China; b Key Laboratory of Ministry of Education of Gastrointestinal Cancer, Fujian Medical University, Fuzhou, China; c Fujian Key Laboratory of Tumor Microbiology, Fujian Medical University, Fuzhou, China

**Keywords:** esophageal hiatal hernias, gastroesophageal reflux disease, randomized clinical trials, research waste, study design

## Abstract

**Background:**

The results of many large randomized clinical trials (RCTs) have transformed clinical practice in gastroesophageal reflux disease (GERD) and esophageal hiatal hernia (HH). However, research waste (i.e., unpublished data, inadequate reporting, or avoidable design limitations) remains a major challenge to evidence-based medicine.

**Method:**

A cross-sectional analysis was conducted to comprehensively review and evaluate RCTs related to GERD and esophageal HH, registered in the ClinicalTrials.gov database between 2003 and 2023. A sample of eligible RCTs was identified by excluding early-stage trials, pediatric studies, and duplicate studies. Publication status was tracked using PubMed and Scopus databases, reporting adequacy was assessed according to the CONSORT guidelines, and design flaws were checked with the help of Cochrane tools. Shortcomings of RCT studies in different regions and intervention types were identified by quantifying RCT conduct, recruitment, reporting adequacy, risk of bias, and guideline citations.

**Results:**

From 2003 to 2023, a total of 182 RCTs were included in the analysis, of which 69.8% (127 trials) were drug-related, and 71.4% of the principal investigators were located in North America and Asia (65 trials [35.7%] in both). Among them, the country with the most RCTs is the United States. RCTs in Asia were under-conducted in “procedure” and “other” types and fewer RCTs were conducted in Europe in “drug” type. RCTs in Oceania and South America were relatively under-conducted in the device and “other.” The study revealed that more than 86.7% of RCTs were characterized by at least one type of research waste. Research waste was associated with the size of the RCT, blinded design, and regional healthcare access and quality index.

**Conclusions:**

This study describes for the first time the characteristics of RCTs for GERD and esophageal HH over the past 20 years and the conduct of various types of RCTs at the continental level. It identifies the burden of research waste and shortcomings in the conduct of RCT programs on each continent, which may provide evidence for the development of rational RCTs and the reduction of waste in the future.

## Introduction

Gastroesophageal reflux disease (GERD) is a common condition affecting adults and children worldwide, with the global prevalence of GERD high and rising^[1–3]^. GERD imposes a significant economic burden in terms of access, diagnosis, cancer surveillance, and treatment^[4–7]^. Esophageal hiatal hernias (HH), an important risk factor of GERD^[8–11]^, are associated with GERD through anatomical and physiological disruption of normal antireflux mechanisms. To improve the prognosis of patients with GERD and HH, numerous randomized clinical trials (RCTs) have been undertaken to identify new and potentially more effective treatments. Over the past 20 years, these efforts have resulted in major advancements in the clinical diagnosis and treatment of GERD and HHs that have significantly improved the quality of life of patients suffering from these afflictions.

Although RCTs provide a high level of evidence and make an important contribution to advancing treatments, research waste is inevitably a major challenge to evidence-based medicine. This means that wasteful RCTs use resources while increasing risks to participants. Chapman *et al*^[12]^ found that 85.2% of surgery-related RCTs had research waste. This waste can occur at any stage of the research cycle. Research waste may ultimately mean that clinical practice guidelines in the relevant field fail to adopt the findings. To date, the issue of research waste in RCTs for GERD and HH has not been explored. Minimizing research waste is critical to ensure that new therapies are appropriately, safely, and effectively applied in clinical practice. This study analyzed the characteristics and components of research waste in GERD and HH RCTs over the past 20 years to identify potential targets for improvement. In addition, we explored whether published RCTs were referenced in guidelines and whether relevant prospective data were reused.

## Methods

The Ethics Committee validated and confirmed that given that this study was a retrospective analysis based on publicly registered RCTs, which did not involve patient recruitment and collection of sensitive information, the routine ethical approval process and the signing of the informed consent form could be exempted. This study strictly complied with the requirements of the guideline of “Strengthening the Reporting of Cohort, Cross-Sectional, and Case-Control Studies in Surgery” (STROCSS)^[13]^.

## Design and data sources

The data for this study was obtained from ClinicalTrials.gov, an authoritative clinical trials registry,^[14]^ which is a publicly available and widely used online platform that compiles detailed registries of clinical studies from around the world^[15–18]^. A comprehensive search of the ClinicalTrials.gov database was conducted on April 1, 2024. The Condition/Disease field was set to “Gastroesophageal Reflux” OR “Hernia, Hiatal”, the Study Status field was limited to completed studies, and the Study Type was specified as interventional. Inclusion criteria encompassed RCTs registered in the database between 2003 and 2023, meeting the study conditions based on a detailed evaluation of trial titles and abstracts. Phase I and Phase II trials were excluded, as the primary goal of these phases is the initial validation of efficacy and safety, which may not follow the routine publication process. Given the unique complexity of pediatric trials at the methodological level and the need for ad hoc review, such studies were not included. In addition, nonrandomized trials, projects not related to GERD and HH, replicated studies, and unpublished trials with a completion date of RCTs later than January 1, 2022, were excluded. This timeframe was selected to ensure that research teams have sufficient time to complete the paper writing, submission, peer review, and editorial process, thereby improving the quality and integrity of published results^[12]^. Independent researchers (A and B) conducted assessments and resolved discrepancies by consensus.

In this study, all included RCTs were ranked by sample size in descending order, with the top 25% classified as “large sample RCTs” due to their higher number of participants. Given the significantly higher prevalence of GERD in North America and Europe compared to other regions^[19-22]^, RCTs were categorized into North American, European, and other continental trials based on the location of the principal investigator (PI). Additionally, recognizing the correlation between the conduct of RCTs and the Healthcare Access and Quality (HAQ) Index across national regions, RCTs were further classified into those conducted in regions with an HAQ index ≥90 and those with an HAQ index <90, according to the HAQ index^[23]^ of the PI’s location. A criterion based on the 75th percentile quartile of the HAQ index of the countries included in the analysis. Based on the World Bank’s income classification^[24]^, this study classifies countries according to their income levels. Since there are few studies on low-middle income and upper-middle income countries, they are combined as nonhigh income countries. They were compared with trials in high-income countries.

## Status of publication

The publication status was determined by searching PubMed and Scopus using the ClinicalTrials.gov identifier (National Clinical Trial [NCT] number), the name of the principal investigator (PI), and relevant keywords associated with the RCT. If the corresponding manuscript was not found on PubMed and Scopus, the corresponding PI was contacted to further confirm the publication status. If no response was received, the RCT was defaulted as unpublished^[12]^. If the full-text manuscript was found in a peer-reviewed journal (print or online), the trial was considered published. The last search was conducted on June 30, 2024.

## Reporting adequacy assessment

The reporting completeness of each article was evaluated through a detailed assessment based on the Consolidated Standards of Reporting Trials (CONSORT) guidelines. Taking into account the different characteristics of pharmacological and nonpharmacological interventions, the CONSORT guidelines design lists 37 and 40 checkpoints, respectively. A study team member (C) was responsible for collecting all relevant publications and their supplementary materials. By using the PDF to Word function of Adobe Acrobat Pro PDF software (Adobe Systems Inc.), he removed author and journal identifiers from the documents and adjusted the text layout as a way to minimize potential bias. The printed documents were then thoroughly reviewed and scored by two independent researchers (D and E) for each piece of literature based on the CONSORT 2010 checklist. After completing the evaluation of each of the three articles, the duo would discuss any disagreements until a consensus was reached^[25]^. An article on a RCT of a pharmacological intervention that meets at least 27 items, or a nonpharmacological RCT that meets at least 30 items, is considered to have met the adequate criteria for reporting. This threshold was set retrospectively according to the median reporting compliance but was a preplanned approach^[12]^.

## Design flaw assessment

Two independent researchers (F and G) used the Cochrane Tool^[26]^. Masked manuscripts were reviewed and assessed for risk of selection bias, performance bias, detection bias, attrition bias, reporting bias, and other biases. The risk of bias for each item was categorized as low, unclear, or high risk. After reviewing every three manuscripts, the investigators discussed and reached a consensus on the discrepancies that existed. In the statistical analysis, items for which the risk of bias was unclear were considered to be at high risk. This is because unclear descriptions of key methods can affect judgments about the ability of an RCT to provide information. In addition, the researchers assessed whether relevant systematic reviews existed or whether there was a need for a systematic review in a new setting. A systematic review was considered to exist only if it was cited in the full text of the manuscript and was considered to justify the need to conduct an RCT. Avoidable design flaws were considered to exist if one of the aforementioned biases was present in the article or if the relevant systematic review was not cited.

## RCTs performed at the continental level

Targeting research funds to a specific region or country would be a more cost-effective means of identifying which types of interventions are comparatively well-studied and which have been neglected in specific parts of the world, and for determining the relative inadequacies of RCT research in a given area. Such an approach would reduce the amount of research waste. Due to the limited number of RCTs categorized as “devices” and “other,” these types were combined and collectively labeled as “other*”.

Our goal was to quantify five dimensions, consisting of (1) the number of RCTs conducted for an intervention, (2) the number of RCTs recruited for an intervention, (3) the reporting adequacy of RCTs for an intervention, (4) the risk of bias in RCTs reported for an intervention, and (5) the number of guidelines cited for RCTs for an intervention. Unless otherwise specified, the RCTs included in this study were default RCTs for GERD and hiatal hernia.

**1. Quantification of the first dimension (number of examples):** (number of RCTs conducted for an intervention in a region/total number of RCTs conducted in the region) × 100%

$$P1=\left(\frac{N_{RCT-intervention}}{N_{RCT-total}}\right)\times 100\%$$

N_RCT-Intervention_: Number of RCTs conducted in a region for a given intervention; N_RCT-total_: Total number of RCTs conducted in the region.

**2. Quantification of the second dimension (number of recruits):** (number of RCTs recruited for intervention in a region/total number of RCTs recruited in a region) × 100%

$$P2=\left(\frac{P_{participants-intervention}}{P_{participants-total}}\right)x100\%$$

P_participants-intervention_: Number of RCTs recruited in a region for a given intervention; P_Participants-total_: Total number of RCTs recruited in the region.

**3. Quantification of the third dimension (number of exceedance items reported by the RCTs based on the CONSORT standard):** The total number of scoring items differed between the drug and nondrug groups at the time of CONSORT scoring, with a total of 37 scoring items in the drug group and 40 scoring items in the nondrug group. Due to this difference, the use of attainment items to quantify the index was deemed unsuitable, and the number of exceeding items was used instead. Thus, if the RCT was designed such that the “drug” was the intervention and its attainment item on the CONSORT form exceeded 27, then it was included in the analysis and the difference between its attainment and 27 was calculated. Similarly, for RCTs with nonpharmacological interventions, if the number of compliance items above the CONSORT form exceeded 30, the RCT was included, and the difference from 30 was calculated. Quantification was accomplished using the following formula: (number of exceeded items on the CONSORT form reported by RCTs in a region for a given intervention/total number of exceeded items reported on the CONSORT form by RCTs in the region) × 100%

$$P3=\left(\frac{C_{CONSORT-intervention}}{C_{CONSORT-total}}\right)x100\%$$

C_CONSORT-intervention_: Number of exceeded items on the CONSORT form reported by RCTs in a region for a given intervention.

C_CONSORT-total_: Total number of exceeded items reported on the CONSORT form by RCTs in the region

**4. Quantification of the fourth dimension (risk of bias in reporting):** (number of items with a low risk of bias reported by the RCTs for a given intervention in a specific region/total number of items with a low risk of bias reported by the RCTs in the region) × 100%

$$P4=\left(\frac{L_{bias-intervention}}{L_{bias-total}}\right)x100\%$$

L_bias-intervention_: Number of items with a low risk of bias reported by RCTs for a given intervention in a specific region. L_bias-total_: Total number of items with a low risk of bias reported by the RCTs in the region

**5. Quantification of the fifth dimension (guide citation):** (number of RCT reports on an intervention in a region cited in the guideline/total number of RCT reports in the region cited in the guidelines) × 100%

$$P5=\left(\frac{G_{guideline-intervention}}{G_{guideline-total}}\right)\times 100\%$$

G_guideline-intervention_: Number of RCT reports for an intervention in a region cited in the guideline. G_guideline-intervention_: Total number of RCT reports in the region cited in the guideline

Team members were then asked to construct a judgment matrix for use in an analytic hierarchy process (AHP). Differences were eliminated through negotiation and consensus to construct the most appropriate judgment matrix (Table 1), which was then used in the AHP to calculate individual item weights. The final weightings were P1 (5.75%), P2 (8.93%), P3 (18.37), P4 (23.38%), and P5 (43.57%).

**Table 1:** The Judgment Matrix of AHP

	P1	P2	P3	P4	P5
P1	1.000	0.500	0.250	0.250	0.200
P2	2.000	1.000	0.333	0.333	0.250
P3	4.000	3.000	1.000	0.500	0.333
P4	4.000	3.000	2.000	1.000	0.333
P5	5.000	4.000	3.000	3.000	1.000

These values were then multiplied by their respective corresponding weights and summed to represent the total RCT score for intervention type S _(Intervention)_:

**S _(Intervention)_ = P1 × 5.75% + P2 × 8.93% + P3 × 18.37% + P4 x 23.38% + P5 × 43.57%**

Finally, the total score of RCTs for the “drug” intervention type, the total score of RCTs for the “procedure” intervention type, and the total score of RCTs for the “other*” intervention type in each region was calculated:

S_Drug_: S_Procedure_: S_Other*._

Additionally, an attempt was made to establish a “universal ratio” to serve as a reference point for the utilization of GERD and HH RCTs across each continent. However, this highlighted several key deficiencies of the current RCT development process. For instance, RCTs with both aspects—that of “drug” and “other*”—were only employed in Asia, Europe, and North America, and RCTs with all three aspects of “procedure,” “drug,” and “other*” were observed in only five countries. To include as much relevant information as possible in the universal ratio, we originally intended to utilize national data as opposed to continent-wide data. Considering that the (drug:procedure:other*) ratio for RCTs may vary by region within a country, a universal ratio derived from data at the national level may be biased by regional differences in the quality of care, and consequently would not be universally applicable. We attempted to minimize this potential problem in two ways: (1) First, the universal ratio was established as a continental goal, rather than requiring less developed countries to achieve the same level as wealthier nations. (2) Second, data from the five countries that utilized RCTs encompassing all three aspects—“procedure,” “drug,” and “other*”—were included in the following algorithm: the S_(Intervention)_ of each country was divided by the corresponding HAQ value to obtain the ADS_(Intervention)_ for each country. The adjusted scores of RCTs for “drug,” “procedure,” and “other*” interventions in each country were calculated and expressed as ADS_(Drug)_, ADS_(Procedure)_, and ADS_(Other*)_, respectively:

$$ADS_{\left(Intervention\right)}=\frac{S_{\left(Intervention\right)}}{HAQ}$$

The ADS_(Intervention)_ values of each country for the same type of intervention RCTs were then summed to obtain ${ADS}_{Drug}^{Total}$, ${ADS}_{Procedure}^{Total}$ and $ADS_{Other^{*}}^{Total}$, and the proportion of $\left({ADS}_{Drug}^{Total}:\;{ADS}_{Procedure}^{Total}\;{:ADS}_{Other^{*}}^{Total}\;\right)$ was calculated, which represented the universal proportion. We then counted the proportion of (S_Drug_: S_Procedure_: S_Other*_) for each continent and compared it with the universal proportion. A system of elimination was applied, wherein a continent falling below and furthest from the universal proportion for a particular aspect was considered relatively underdeveloped in that area.

## Whether referenced in guidelines and reuse of prospective data

Excluding RCTs published in the last year (2023), for the remaining publicly published RCTs, our initial step was to Google Scholar, a scholarly search engine^[27]^ that tracks all research literature that cites RCTs. Next, two unaffiliated researchers (H & I) personally reviewed this literature, and their task was to screen it for the presence of treatment or practice guidelines. In addition, we assessed whether these follow-up studies utilized prospective data from the original RCTs for post hoc profiling, i.e., whether the data from the original RCTs were re-analyzed to yield outcomes other than the predefined primary and secondary study endpoints^[28–30]^.

## Outcome

The main objective of the study is to characterize the clinical research trials conducted over the past two decades and to provide insights into the phenomenon of so-called “research waste” including nonpublication, inadequate reporting, and avoidable design flaws. While at the same time, it also analyzes the conduct of RCTs for different interventions on different continents. In addition, this study examines whether published RCTs are cited in guidelines and whether prospective data are reused, as published RCTs are a prerequisite for assessing reporting adequacy and design flaws. All RCTs completed after January 1, 2022, and not published were excluded from the analysis.

## Statistical analysis

Differences in categorical variables between groups were compared using χ^2^ or Fisher test (if the sample size was less than 5)^[31]^. Simple and multivariate logistic regression models were used to identify independent risk factors associated with research waste. Variables with *P* < 0.05 in simple analyses were subsequently included in multivariate analyses. All statistical analyses were performed using SPSS statistical software for Windows, version 18.0 (IBM), and R statistical software, version 4.0.2 (R Project for Statistical Computing). *P*-values < 0.05 were considered statistically significant, and all tests were two-sided. The data were analyzed in July 2024, and all tests were two-sided.

## Results

### RCT development on a global scale

**Figure 1.**
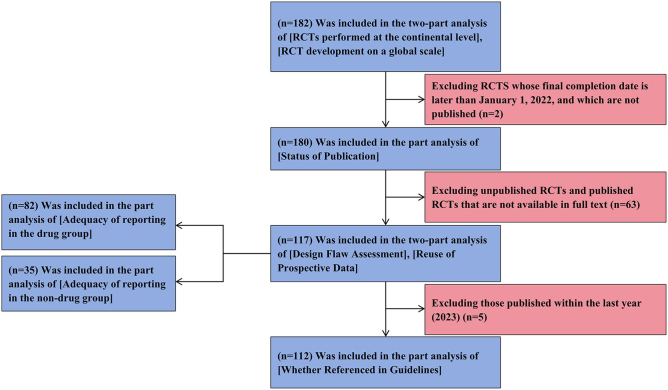
Flow diagram.

**Figure 2.**
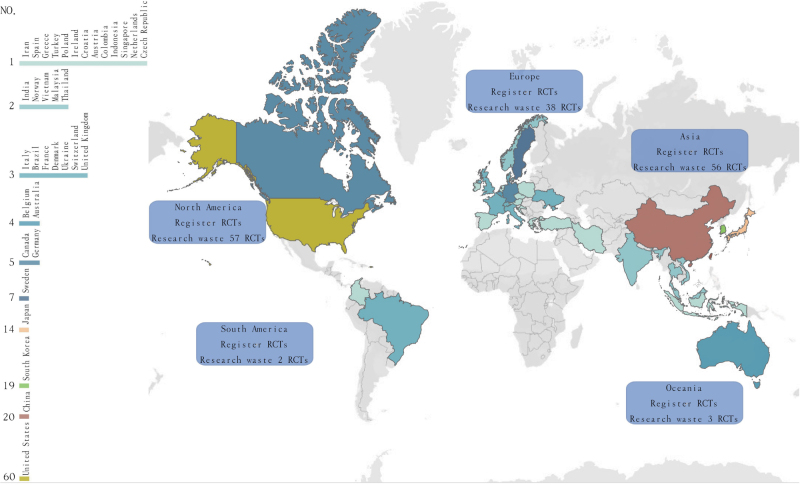
RCT development on a global scale.

From 2003 to 2023, a total of 202 clinical trials that met the inclusion criteria were retrieved. A total of 182 RCTs were included in the analysis after excluding eight nonrandomized clinical trials, one pediatric trial, three trials not related to GERD or HH, and eight duplicates (Fig. 1). A total of 69.8% of the studies were drug-related (127 trials). A total of 71.4% of the principal investigators were located in North America and Asia (65 [35.7%] in both). The country with the most trials related to GERD and HH was the United States (60 trials [33.0%]) (Fig. 2). There were 100 multicenter clinical trials in the study (54.9%). The median (interquartile spacing) sample size of RCTs was 139 (58–337). Therefore, we defined RCTs with sample sizes greater than 350 individuals as large RCTs. In the included sample, the number of RCTs funded internally and externally was the same, with 91 (50.0%). Other relevant information is provided in Table 2.

**Table 2:** Characteristics of All RCTs

Characteristic	RCTs, NO. (%) N = 182
Time of registration	
2003–2008	76 (41.8)
2009–2013	44 (24.2)
2014–2018	29 (15.9)
2019–2023	33 (18.1)
Intervention	
Drug	127 (69.8)
Procedure	34 (18.7)
Device	12 (6.6)
Other	9 (4.9)
Primary purpose	
Treatment	158 (86.8)
Prevent	1 (0.5)
Support care	1 (0.5)
Other	22 (12.1)
Intervention model	
Parallel	152 (83.5)
Factorial	2 (1.1)
Crossover	28 (15.4)
Arm	
2	151 (83.0)
3	22 (12.1)
≥4	6 (3.3)
Missing	3 (1.6)
Blinding	
None or open label	49 (26.9)
Single	17 (9.3)
Double	66 (36.3)
Triple	19 (10.4)
Quadruple	31 (17.0)
Recruitment	
Monocentric	76 (41.8)
Multicenter	100 (54.9)
Missing	6 (3.3)
Funder type	
None or departmental	91 (50.0)
Industry or other external	91 (50.0)
Region of PI	
North America	65 (35.7)
Europe	44 (24.2)
Asia	65 (35.7)
South America	4 (2.2)
Oceania	4 (2.2)
Region of PI	
HAQ<90	164 (90.1)
HAQ≥90	18 (9.9)
Region of PI	
High Income	144 (79.1)
Nonhigh income	38 (20.9)
Region of PI	
High SDI	138 (75.8)
Nonhigh SDI	44 (24.2)

**Figure 3.**
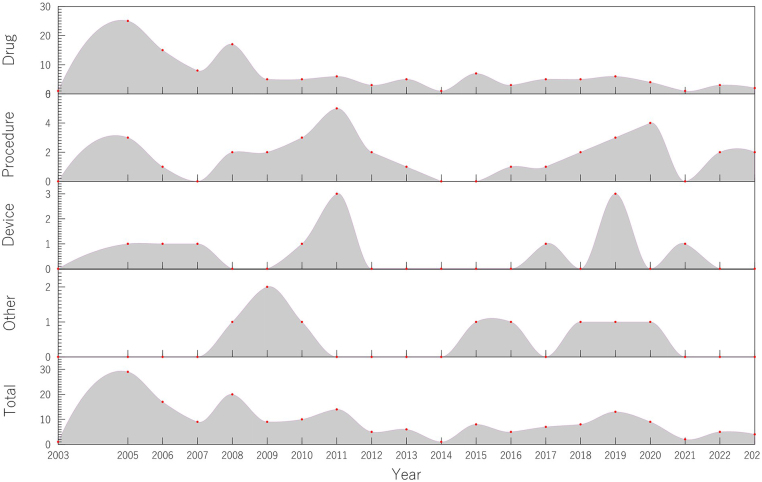
Development of RCT.

**Figure 4.**
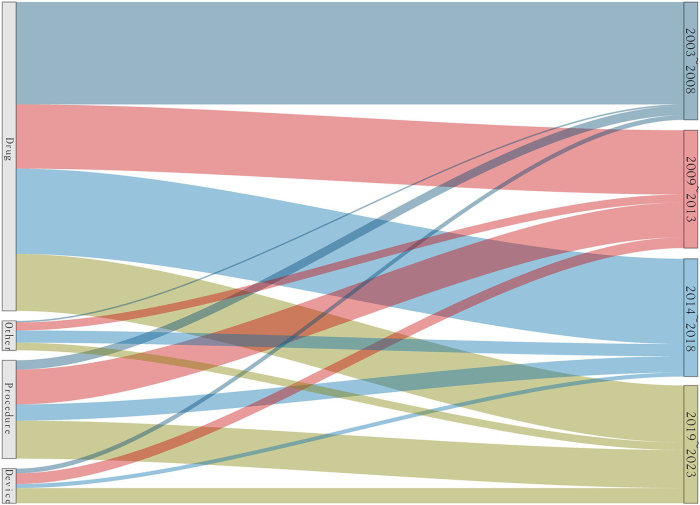
The proportion of different types of RCTS.

The number of RCTs of GERD and HH started with only 1 study in 2003 and reached a peak of 29 in 2005. Since then, the number of studies has begun to ebb and flow. Starting in 2015, the number of studies seems to have stabilized, albeit at a lower number compared with the previous peak, remaining between 2 and 13 studies per year. From 2003 to 2023, different types of RCTs showed large fluctuations (Fig. 3). Over the 20 years, the number of RCTs in the “drug” category consistently accounted for the largest share. There are fewer RCTs of “procedure” and “device” intervention types than those of “drug” intervention. The “other” category has the lowest number of RCTs (Fig. 4).

### Nonpublication

Overall, a total of 129 registered RCTs (71.7%) were published in peer-reviewed journals, of which 117 (65.0%) were published and available for review in full. Fifty-one (28.3%) were unpublished. Unpublished RCTs are more likely to be of the type of grant funded by industry or other external compared to published RCTs (32 trials [62.75%] vs. 57 trials [44.19%]; *P* = 0.025) and were more likely to originate from areas with a HAQ index of less than 90 (50 trials [98.04%] vs. 112 trials [86.82%]; *P* = 0.024) (Table 3). Further analysis found that RCTs funded by Industry or other external sources were more unlikely to be published (odds ratio [OR], 0.470; 95% CI, 0.242 to 0.915; *P* = 0.026) (Table 4).

**Table 3:** Characteristics of RCTs According to Publication Status

Characteristic	Not published, No. (%)	Published, No. (%)	Total, No. (%)	*P*
Funder type				
None or departmental	19 (37.25)	72 (55.81)	91 (50.56)	0.025*
Industry or other external	32 (62.75)	57 (44.19)	89 (49.44)	
Intervention				
Procedure	5 (9.80)	28 (21.71)	33 (18.33)	0.273
Drug	39 (76.47)	87 (67.44)	126 (70.00)	
Device	4 (7.84)	8 (6.20)	12 (6.67)	
Other	3 (5.88)	6 (4.65)	9 (5.00)	
Recruitment				
Single center	21 (41.18)	53 (41.09)	74 (41.11)	0.956
Multicenter	28 (54.90)	72 (55.81)	100 (55.56)	
Missing	2 (3.92)	4 (3.10)	6 (3.33)	
Arm				
2	44 (86.27)	105 (81.40)	149 (82.78)	0.243
3	3 (5.88)	19 (14.73)	22 (12.22)	
≥4	1 (1.96)	5 (3.88)	6 (3.33)	
Missing	3 (5.88)	0 (0.00)	3 (1.67)	
Intervention model				
Crossover	9 (17.65)	19 (14.73)	28 (15.56)	0.828
Parallel	42 (82.35)	108 (83.72)	150 (83.33)	
Factorial	0 (0.00)	2 (1.55)	2 (1.11)	
Blinding				
None or open label	12 (23.53)	37 (28.68)	49 (27.22)	0.416
Single	3 (5.88)	14 (10.85)	17 (9.44)	
Double or more	36 (70.59)	78 (60.47)	114 (63.33)	
Primary purpose				
Prevent	0 (0.00)	1 (0.78)	1 (0.56)	0.163
Other	3 (5.88)	18 (13.95)	21 (11.67)	
Supportcare	1 (1.96)	0 (0.00)	1 (0.56)	
Treatment	47 (92.16)	110 (85.27)	157 (87.22)	
No. of participants				
<350	42 (82.35)	93 (72.09)	135 (75.00)	0.152
≥350	9 (17.65)	36 (27.91)	45 (25.00)	
PI region				
<90	50 (98.04)	112 (86.82)	162 (90.00)	0.024*
≥90	1 (1.96)	17 (13.18)	18 (10.00)	
PI region				
North America	17 (33.33)	47 (36.43)	64 (35.56)	0.670
Europe	11 (21.57)	33 (25.58)	44 (24.44)	
Other	23 (45.10)	49 (37.98)	72 (40.00)	
PI region				
Nonhigh SDI	13 (25.49)	30 (23.26)	43 (23.89)	0.751
High SDI	38 (74.51)	99 (76.74)	137 (76.11)	
PI region				
High income	40 (78.43)	103 (79.84)	143 (79.44)	0.833
Nonhigh income	11 (21.57)	26 (20.16)	37 (20.56)	
Time of registration				
2003–2013.06	33 (64.71)	84 (65.12)	117 (65.00)	0.959
2013.06–2023	18 (35.29)	45 (34.88)	63 (35.00)	

* *P* < 0.05

**Table 4:** Adjusted Logistic Regression Analysis of Association of Key Study Characteristics with Publication Status

Characteristic	Univariate analysis		Multivariate analysis	
	OR (95% CI)	*P* value	OR (95% CI)	*P* value
Time of registration				
2003–2013.06	1			
2013.06–2023	0.982 (0.498–1.937)	0.959		
Intervention				
Nonpharmacological related	1			
Pharmacological related	0.637 (0.303–1.342)	0.236		
Arm				
2	1			
3	2.654 (0.747–9.427)	0.131		
≥4	2.095 (0.238–18.455)	0.505		
Blinding				
None or open label	1			
Single	1.514 (0.371–6.179)	0.564		
Double or more	0.703 (0.328–1.505)	0.364		
Funder type				
None or departmental	1			
Industry or other external	0.470 (0.242–0.915)	0.026*		
Recruitment				
Single center	1			
Multicenter	1.019 (0.523–1.987)	0.956		
No. of participants				
<350	1			
≥350	1.806 (0.799–4.086)	0.156		
PI region				
Nonhigh SDI	1			
High SDI	1.129 (0.533–2.391)	0.751		
PI region				
<90	1			
≥90	7.589 (0.983–58.609)	0.052		
PI region				
High income	1			
Nonhigh income	0.918 (0.415–2.030)	0.833		
PI region				
North America	1			
Europe	1.085 (0.450–2.614)	0.856		
Other	0.771 (0.366–1.621)	0.492		

* *P* < 0.05

### Adequacy of reporting

The 117 published RCTs were scored according to the CONSORT checklist. A total of 62 randomized controlled studies (53.0%) were judged to be adequately reported. Of the 82 RCTs of pharmacological interventions, 43 (52.4%) were adequately reported, and these RCTs were more likely to have originated from regions other than North America and Europe (25 trials [58.14%] vs. 13 trials [33.33%]; *P* = 0.050) and to have had treatment as the primary purpose of the trial (42 trials [97.67%] vs. 32 trials [82.05%]; *P* = 0.028). Compared with fully reported RCTs, underreported RCTs are more likely to adopt the crossover design approach (10 trials [25.64%] vs. 3 trials [6.98%]; *P* = 0.030) (Table 5). The most notable deficiencies in reporting were the failure to provide access to trial protocols (present in 2.4% of RCTs), discussion of the applicability of trial results (20.7%), and description of the type of randomization (25.6%). Of the 35 studies using nonpharmacological interventions, 19 (54.3%) RCTs were adequately reported. Underreported RCTs were more likely to have been enrolled by June 2013 than adequately reported RCTs (13 trials [81.25%] vs. 9 trials [47.37%]; *P* = 0.039) (Table 6). The most commonly reported deficiencies were the provision of access to the trial protocol (present in 5.7% of RCTs), description of the details of assessing or enhancing adherence to the regimen by care attesters (8.6%), discussion of the applicability of the trial results (34.3%), and description of the type of randomization (34.3%). (Table 7).

**Table 5:** Characteristics of Randomized Clinical Trials by Reporting Adequacy (Drug Group)

Characteristic	Adequate reporting, No. (%)	Inadequate reporting, No. (%)	Total, No. (%)	*P*
Time of registration				
2003–2013.06	29 (67.44)	30 (76.92)	59 (71.95)	0.340
2013.06–2023	14 (32.56)	9 (23.08)	23 (28.05)	
Primary purpose				
Treatment	42 (97.67)	32 (82.05)	74 (90.24)	0.028*
Other	1 (2.33)	6 (15.38)	7 (8.54)	
Prevent	0 (0.00)	1 (2.56)	1 (1.22)	
Intervention model				
Crossover	3 (6.98)	10 (25.64)	13 (15.85)	0.030*
Parallel	39 (90.70)	29 (74.36)	68 (82.93)	
Factorial	1 (2.33)	0 (0.00)	1 (1.22)	
Arm				
2	31 (72.09)	31 (79.49)	62 (75.61)	0.371
3	9 (20.93)	8 (20.51)	17 (20.73)	
≥4	3 (6.98)	0 (0.00)	3 (3.66)	
Blinding				
None or open label	11 (25.58)	10 (25.64)	21 (25.61)	0.923
Single	1 (2.33)	2 (5.13)	3 (3.66)	
Double or more	31 (72.09)	27 (69.23)	58 (70.73)	
Funder type				
None or departmental	17 (39.53)	16(41.03)	33(40.24)	0.891
Industry or other external	26 (60.47)	23(58.97)	49(59.76)	
Recruitment				
Single center	15 (34.88)	12 (30.77)	27 (32.93)	0.817
Multicenter	28 (65.12)	25 (64.10)	53 (64.63)	
Missing	0 (0.00)	2 (5.13)	2 (2.44)	
No. of participants				
<350	27 (62.79)	22 (56.41)	49 (59.76)	0.556
≥350	16 (37.21)	17 (43.59)	33 (40.24)	
PI region				
Nonhigh SDI	12 (27.91)	7 (17.95)	19 (23.17)	0.286
High SDI	31 (72.09)	32 (82.05)	63 (76.83)	
PI region				
<90	40 (93.02)	35 (89.74)	75 (91.46)	0.703
≥90	3 (6.98)	4 (10.26)	7 (8.54)	
PI region				
High income	31 (72.09)	34 (87.18)	65 (79.27)	0.092
Nonhigh income	12 (27.91)	5 (12.82)	17 (20.73)	
PI region				
North America	13 (30.23)	15 (38.46)	28 (34.15)	0.050*
Europe	5 (11.63)	11 (28.21)	16 (19.51)	
Other	25 (58.14)	13 (33.33)	38 (46.34)	

* *P* < 0.05

**Table 6:** Characteristics of RCT by Reporting Adequacy (Nondrug Group)

Characteristic	Adequate reporting, No. (%)	Inadequate reporting, No. (%)	Total, No. (%)	P
Time of registration				
2003–2013.06	9 (47.37)	13 (81.25)	22 (62.86)	0.039*
2013.06–2023	10 (52.63)	3 (18.75)	13 (37.14)	
Primary purpose				
Treatment	15 (78.95)	10 (62.50)	25 (71.43)	0.454
Other	4 (21.05)	6 (37.50)	10 (28.57)	
Intervention model				
Crossover	2 (10.53)	4 (25.00)	6 (17.14)	0.379
Parallel	17 (89.47)	12 (75.00)	29 (82.86)	
Arm				
2	16 (84.21)	16 (100.00)	32 (91.43)	0.488
3	1 (5.26)	0 (0.00)	1 (2.86)	
≥4	2 (10.53)	0 (0.00)	2 (5.71)	
Blinding				
None or open label	5 (26.32)	8 (50.00)	13 (37.14)	0.435
Single	6 (31.58)	3 (18.75)	9 (25.71)	
Double or more	8 (42.11)	5 (31.25)	13 (37.14)	
Funder type				
None or departmental	16 (84.21)	13 (81.25)	29 (82.86)	1.000
Industry or other external	3 (15.79)	3 (18.75)	6 (17.14)	
Recruitment				
Single center	10 (52.63)	9 (56.25)	19 (54.29)	0.968
Multicenter	8 (42.11)	7 (43.75)	15 (42.86)	
Missing	1 (5.26)	0 (0.00)	1 (2.86)	
No. of participants				
<350	17 (89.47)	15 (93.75)	32 (91.43)	1.000
≥350	2 (10.53)	1 (6.25)	3 (8.57)	
PI region				
Nonhigh SDI	4 (21.05)	2 (12.50)	6 (17.14)	0.666
High SDI	15 (78.95)	14 (87.50)	29 (82.86)	
PI region				
<90	11 (57.89)	14 (87.50)	25 (71.43)	0.071
≥90	8 (42.11)	2 (12.50)	10 (28.57)	
PI region				
High income	16 (84.21)	15 (93.75)	31 (88.57)	0.608
Nonhigh income	3 (15.79)	1 (6.25)	4 (11.43)	
PI region				
North America	7 (36.84)	7 (43.75)	14 (40.00)	0.317
Europe	9 (47.37)	4 (25.00)	13 (37.14)	
Other	3 (15.79)	5 (31.25)	8 (22.86)	

* *P* < 0.05

**Table 7:** Compliance with Items of the CONSORT 2010 Checklist

CONSORT item		Pharmacological *n* = 82	NPI *n* = 35
1a	Identification as a randomised trial in the title	65 (79.3%)	34 (97. 1%)
1b	Structured summary of trial design, methods, results, and conclusions	56 (68.3%)	19 (54.3%)
2a	Scientific background and explanation of rationale	82 (100.0%)	35 (100.0%)
2b	Specific objectives or hypotheses	80 (97.6%)	34 (97. 1%)
3a	Description of trial design (such as parallel, factorial) including allocation ratio	48 (58.5%)	23 (65.7%)
3b*	Important changes to methods after trial commencement (such as eligibility criteria), with reasons	-	-
4a	Eligibility criteria for participants	81 (98.8%)	34 (97. 1%)
4b	Settings and locations where the data were collected	55 (67.1%)	31 (88.6%)
5	The interventions for each group with sufficient details to allow replication	77 (93.9%)	33 (94.3%)
5A**	Description of the components of the interventions and, if applicable, the procedure for individualizing treatment	N/A	25 (71.4%)
5B**	Details of how the interventions were standardized	N/A	28 (80.0%)
5C**	Details of how the adherence of care provers with the protocol was assessed or enhanced	N/A	3 (8.6%)
6a	Completely defined pre-specified primary and secondary outcome measures	71 (86.6%)	28 (80.0%)
6b*	Any changes to trial outcomes after the trial commenced, with reasons	-	-
7a	How sample size was determined	52 (63.4%)	25 (71.4%)
7b*	When applicable, explanation of any interim analyses and stopping guidelines	-	-
8a	Method used to generate the random allocation sequence	43 (52.4%)	26 (74.3%)
8b	Type of randomization; details of any restriction (such as blocking and block size)	21 (25.6%)	12 (34.3%)
9	Mechanism used to implement the random allocation sequence	29 (35.4%)	18 (51.4%)
10	Who generated the random allocation sequence, enrolled participants, and assigned participants to interventions	23 (28.0%)	13 (37. 1%)
11a	If done, who was blinded after assignment to interventions and how	30 (36.6%)	14 (40.0%)
11b*	If relevant, description of the similarity of interventions	-	-
12a	Statistical methods used to compare groups for primary and secondary outcomes	80 (97.6%)	34 (97. 1%)
12b*	Methods for additional analyses, such as subgroup analyses and adjusted analyses	-	-
13a	The numbers of participants who were randomised, received treatment, and analysed for the primary outcome	76 (92.7%)	32 (91.4%)
13b	For each group, losses and exclusions after randomisation, together with reasons	62 (75.6%)	21 (60.0%)
14a	Dates defining the periods of recruitment and follow-up	35 (42.7%)	18 (51.4%)
14b*	Why the trial ended or was stopped	-	-
15	A table showing baseline demographic and clinical characteristics for each group	70 (85.4%)	24 (68.6%)
16	For each group, number of participants analyzed and whether the analysis was by original assigned groups	69 (84. 1%)	30 (85.7%)
17a	For each primary and secondary outcome, results for each group, and the estimated effect size and its precision	64 (78.0%)	27 (77. 1%)
17b	For binary outcomes, presentation of both absolute and relative effect sizes is recommended	28 (34. 1%)	14 (40.0%)
18*	Results of any other analyses performed, including subgroup analyses and adjusted analyses	-	-
19	All important harms or unintended effects in each group	61 (74.4%)	25 (71.4%)
20	Trial limitations, addressing sources of potential bias, imprecision, and, if relevant, multiplicity of analyses	37 (45. 1%)	23 (65.7%)
21	Generalizability (external validity, applicability) of the trial findings	17 (20.7%)	12 (34.3%)
22	Interpretation consistent with results, balancing benefits and harms, and considering other relevant evidence	55 (67.1%)	27 (77. 1%)
23	Registration number and name of trial registry	62 (75.6%)	28 (80.0%)
24	Where the full trial protocol can be accessed, if available	2 (2.4%)	2 (5.7%)
25	Sources of funding and other support (such as supply of drugs), role of funders	60 (73. 2%)	27 (77. 1%)

* indicates a conditional item for which not all manuscripts were scored

** items relate to nonpharmacological (NPI) RCTs only

### Design limitation

**Figure 5.**
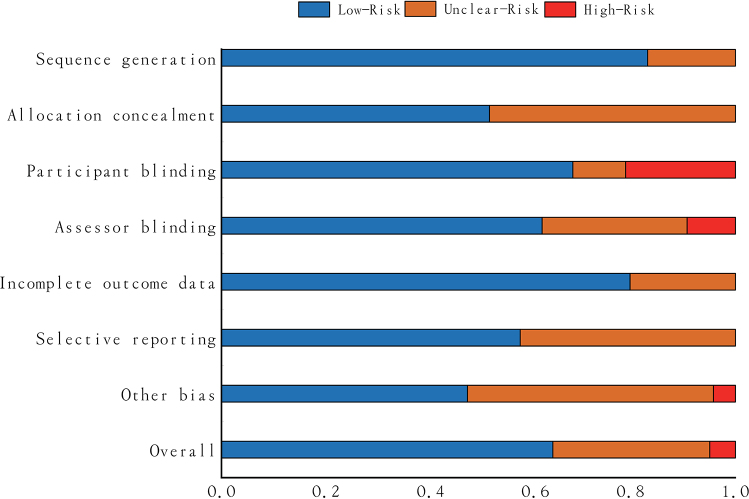
Bias risk.

Of the 117 published studies, 60 studies (51.3%) did not cite a systematic review in the body of the text, and 65 studies (55.6%) had one or more features indicating a high or unclear risk of bias. The most common factors associated with the risk of bias were other bias (61 trials [52.1%]), randomized sequence assignment concealment (56 trials [47.9%]), and selective reporting (49 trials [41.9%]) (Fig. 5). Considering all these factors, 90 RCTs (76.9%) were judged to have avoidable design flaws. RCTs with no avoidable design flaws were more likely to have a double-blind or even multi-blind design compared with these trials (22 trials [81.48%] vs. 49 trials [54.44%]; *P* = 0.025) (Table 8).

**Table 8:** Characteristics of Randomized Clinical Trials by Presence of Avoidable Design Flaws

Characteristic	Absence of design flaw, No. (%)	Presence of design flaw, No. (%)	Total, No. (%)	*P*
Time of registration				
2003–2013.06	15 (55.56)	66 (73.33)	81 (69.23)	0.079
2013.06–2023	12 (44.44)	24 (26.67)	36 (30.77)	
Primary purpose				
Treatment	25 (92.59)	74 (82.22)	99 (84.62)	0.505
Other	2 (7.41)	15 (16.67)	17 (14.53)	
Prevent	0 (0.00)	1 (1.11)	1 (0.85)	
Intervention				
Pharmacological	19 (70.37)	63 (70.00)	82 (70.09)	0.971
Nonpharmacological	8 (29.63)	27 (30.00)	35 (29.91)	
Intervention model				
Crossover	2 (7.41)	17 (18.89)	19 (16.24)	0.108
Parallel	24 (88.89)	73 (81.11)	97 (82.91)	
Factorial	1 (3.70)	0 (0.00)	1 (0.85)	
Arm				
2	21 (77.78)	73 (81.11)	94 (80.34)	0.671
3	4 (14.81)	14 (15.56)	18 (15.38)	
≥4	2 (7.41)	3 (3.33)	5 (4.27)	
Blinding				
None or open label	3 (11.11)	31 (34.44)	34 (29.06)	0.025*
Single	2 (7.41)	10 (11.11)	12 (10.26)	
Double or more	22 (81.48)	49 (54.44)	71 (60.68)	
Funder type				
None or departmental	16 (59.26)	46 (51.11)	62 (52.99)	0.457
Industry or other external	11 (40.74)	44 (48.89)	55 (47.01)	
Recruitment				
Single center	11 (40.74)	35 (38.89)	46 (39.32)	0.674
Multicenter	14 (51.85)	54 (60.00)	68 (58.12)	
Missing	2 (7.41)	1 (1.11)	3 (2.56)	
No. of participants				
<350	17 (62.96)	64 (71.11)	81 (69.23)	0.421
≥350	10 (37.04)	26 (28.89)	36 (30.77)	
PI region				
Nonhigh SDI	8 (29.63)	17 (18.89)	25 (21.37)	0.232
High SDI	19 (70.37)	73 (81.11)	92 (78.63)	
PI region				
<90	21 (77.78)	79 (87.78)	100 (85.47)	0.218
≥90	6 (22.22)	11 (12.22)	17 (14.53)	
PI region				
High income	21 (77.78)	75 (83.33)	96 (82.05)	0.570
Nonhigh income	6 (22.22)	15 (16.67)	21 (17.95)	
PI region				
North America	8 (29.63)	34 (37.78)	42 (35.90)	0.693
Europe	8 (29.63)	21 (23.33)	29 (24.79)	
Other	11 (40.74)	35 (38.89)	46 (39.32)	

* *P* < 0.05

### Research waste

Considering a combination of publication status, adequate reporting, and avoidable design flaws, 156 of the 180 RCTs (86.7%) were characterized by 1 or more research wastes. Compared with the 24 RCTs with no research waste, these RCTs were more likely to be nonlarge (number <350) RCTs (121 trials [77.56%] vs. 14 [58.33%]; *P* = 0.043) and to originate from countries with an HAQ index of <90 (144 trials [92.31%] vs. 18 trials [75.00%]; *P* = 0.019) (Table 9). In further analyses, double-blind and multi-blind designs (odds ratio [OR], 0.193; 95% CI, 0.042–0.894; *P* = 0.035), and trials conducted in areas with a HAQ index ≥90 ([OR], 0.236; 95% CI, 0.073–0.765; *P* = 0.016) were associated with lower odds of research waste (Table 10).

**Table 9:** Characteristics of RCTs according to Research Waste

Characteristic	Without research waste, No. (%)	With research waste, No. (%)	Total, No. (%)	*P*
Funder type				
None or departmental	13 (54.17)	78 (50.00)	91 (50.56)	0.704
Industry or other external	11 (45.83)	78 (50.00)	89 (49.44)	
Intervention				
Procedure	5 (20.83)	28 (17.95)	33 (18.33)	0.740
Drug	17 (70.83)	109 (69.87)	126 (70.00)	
Device	2 (8.33)	10 (6.41)	12 (6.67)	
Other	0 (0.00)	9 (5.77)	9 (5.00)	
Recruitment				
Single center	10 (41.67)	64 (41.03)	74 (41.11)	0.921
Multicenter	13 (54.17)	87 (55.77)	100 (55.56)	
Missing	1 (4.17)	5 (3.21)	6 (3.33)	
Arm				
2	18 (75.00)	131 (83.97)	149 (82.78)	0.185
3	4 (16.67)	18 (11.54)	22 (12.22)	
≥4	2 (8.33)	4 (2.56)	6 (3.33)	
Missing	0 (0.00)	3 (1.92)	3 (1.67)	
Intervention model				
Crossover	1 (4.17)	27 (17.31)	28 (15.56)	0.073
Parallel	22 (91.67)	128 (82.05)	150 (83.33)	
Factorial	1 (4.17)	1 (0.64)	2 (1.11)	
Blinding				
None or open label	2 (8.33)	47 (30.13)	49 (27.22)	0.059
Single	2 (8.33)	15 (9.62)	17 (9.44)	
Double or more	20 (83.33)	94 (60.26)	114 (63.33)	
Primary purpose				
Treatment	22 (91.67)	135 (86.54)	157 (87.22)	0.808
Other	2 (8.33)	19 (12.18)	21 (11.67)	
Supportcare	0 (0.00)	1 (0.64)	1 (0.56)	
Prevent	0 (0.00)	1 (0.64)	1 (0.56)	
No. of participants				
<350	14 (58.33)	121 (77.56)	135 (75.00)	0.043*
≥350	10 (41.67)	35 (22.44)	45 (25.00)	
PI region				
<90	18 (75.00)	144 (92.31)	162 (90.00)	0.019*
≥90	6 (25.00)	12 (7.69)	18 (10.00)	
PI region				
North America	7 (29.17)	57 (36.54)	64 (35.56)	0.757
Europe	6 (25.00)	38 (24.36)	44 (24.44)	
Other	11 (45.83)	61 (39.10)	72 (40.00)	
PI region				
Nonhigh SDI	7 (29.17)	36 (23.08)	43 (23.89)	0.515
High SDI	17 (70.83)	120 (76.92)	137 (76.11)	
PI region				
High income	18 (75.00)	125 (80.13)	143 (79.44)	0.590
Nonhigh income	6 (25.00)	31 (19.87)	37 (20.56)	
Time of registration				
2003 ~ 2013.06	14 (58.33)	103 (66.03)	117 (65.00)	0.462
2013.06 ~ 2023	10 (41.67)	53 (33.97)	63 (35.00)	

* *P* < 0.05

**Table 10:** Adjusted Logistic Regression Analysis of Association of Key Study Characteristics with Research Waste

Characteristic	Univariate analysis		Multivariate analysis	
	OR (95% CI)	*P*-value	OR (95% CI)	*P*-value
Time of registration				
2003–2013.06	1			
2013.06–2023	0.720 (0.300–1.731)	0.463		
Intervention				
Nonpharmacological related	1			
Pharmacological related	0.955 (0.371–2.455)	0.924		
Arm				
2	1			
3	0.618 (0.188–2.033)	0.429		
≥4	0.275 (0.047–1.609)	0.152		
Blinding				
None or open label	1			
Single	0.319 (0.041–2.465)	0.274	0.335 (0.040–2.812)	0.313
Double or more	0.200(0.045–0.892)	0.035*	0.193 (0.042–0.894)	0.035*
Funder type				
None or departmental	1			
Industry or other external	1.182 (0.499–2.799)	0.704		
Recruitment				
Single center	1			
Multicenter	1.046 (0.431–2.535)	0.921		
No. of participants				
<350	1			
≥350	0.405 (0.166–0.991)	0.048*	0.445(0.182–1.151)	0.095
PI region				
Nonhigh SDI	1			
High SDI	1.373 (0.528–3.569)	0.516		
PI region				
<90	1			
≥90	0.250 (0.084–0.748)	0.013*	0.236 (0.073–0.765)	0.016*
PI region				
High income	1			
Nonhigh income	0.744 (0.273–2.031)	0.564		
PI region				
North America	1			
Europe	0.778 (0.243–2.494)	0.672		
Other	0.681 (0.247–1.877)	0.458		
				

* *P* < 0.05

### RCTs performed at the Continental Level

**Figure 6.**
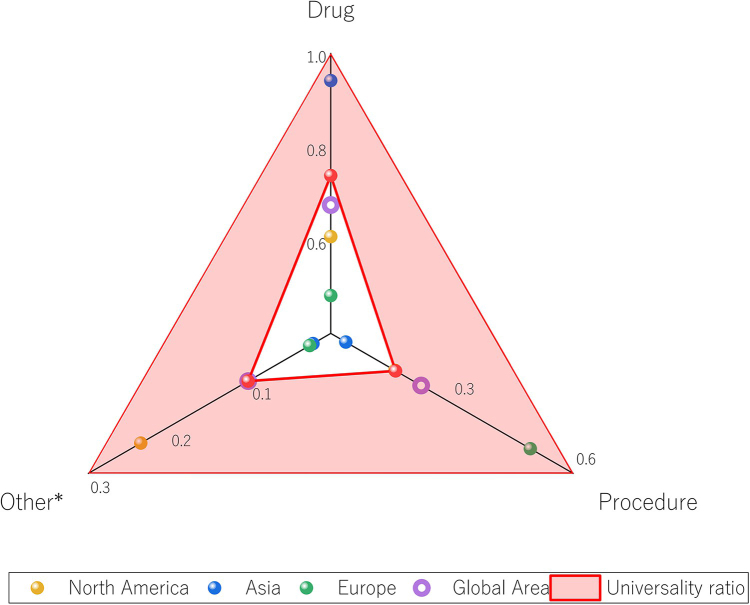
Development of different types of RCTS.

After the above method, we finally obtained a universal ratio of (7.22:1.57:1). “Procedure” and “other*” types of RCTs in Asia may have a relatively underdeveloped situation, which is the short board of GERD and esophageal HH RCTs in the Asian region. “Drug” type of RCTs in Europe may have a relatively underdeveloped situation, which is the short board of the European region for conducting regional RCTs for GERD and HH (Fig. 6). It is important to note that in the current study, Oceania and South America involving GERD and HH RCTs were 0 for “other*”, pending further development.

### Referenced in guidelines and reuse of prospective data

We excluded five RCTs published in the most recent year (2023) and found that 36 trials (32.1%) were cited in the corresponding guidelines, especially multicenter trials (27 trials [75.00%] vs. 38 [50.00%], *P* = 0.022) and those more likely to originate from countries with an HAQ index of ≥90 (9 trials [25.00%] vs. 8 [10.53%], *P* = 0.046) (Table 11). Multicenter RCTs were more likely to be cited in guidelines ([OR], 2.763; 95% CI, 1.143–6.683; *P* = 0.024) (Table 12). In addition, total prospective data from a total of 24 RCTs (20.5%) were reused, but there were no significant differences between groups (Table 13).

**Table 11:** Characteristics of Randomized Clinical Trials by Presence of Guideline Citation

Characteristic	Absence of citing by guidelines, No. (%)	Presence of citing by guidelines, No. (%)	Total, No. (%)	*P*
Time of registration				
2003 ~ 2013.06	55 (72.37)	26 (72.22)	81 (72.32)	0.987
2013.06 ~ 2023	21 (27.63)	10 (27.78)	31 (27.68)	
Primary purpose				
Treatment	62 (81.58)	32 (88.89)	94 (83.93)	0.141
Other	14 (18.42)	3 (8.33)	17 (15.18)	
Prevent	0 (0.00)	1 (2.78)	1 (0.89)	
Intervention				
Pharmacological	56 (73.68)	22 (61.11)	78 (69.64)	0.177
Nonpharmacological	20 (26.32)	14 (38.89)	34 (30.36)	
Intervention model				
Crossover	15 (19.74)	4 (11.11)	19 (16.96)	0.517
Parallel	60 (78.95)	32 (88.89)	92 (82.14)	
Factorial	1 (1.32)	0 (0.00)	1 (0.89)	
Arm				
2	65 (85.53)	25 (69.44)	90 (80.36)	0.097
3	8 (10.53)	9 (25.00)	17 (15.18)	
≥4	3 (3.95)	2 (5.56)	5 (4.46)	
Blinding				
None or open label	22 (28.95)	12 (33.33)	34 (30.36)	0.442
Single	6 (7.89)	5 (13.89)	11 (9.82)	
Double or more	48 (63.16)	19 (52.78)	67 (59.82)	
Funder type				
None or departmental	43 (56.58)	17 (47.22)	60 (53.57)	0.354
Industry or other external	33 (43.42)	19 (52.78)	52 (46.43)	
Recruitment				
Single center	35 (46.05)	9 (25.00)	44 (39.29)	0.022*
Multicenter	38 (50.00)	27 (75.00)	65 (58.04)	
Missing	3 (3.95)	0 (0.00)	3 (2.68)	
No. of participants				
<350	55 (72.37)	22 (61.11)	77 (68.75)	0.230
≥350	21 (27.63)	14 (38.89)	35 (31.25)	
PI region				
Nonhigh SDI	20 (26.31)	4 (11.11)	24 (21.43)	0.067
High SDI	56 (73.68)	32 (88.89)	88 (78.57)	
PI region				
<90	68 (89.47)	27 (75.00)	95 (84.82)	0.046*
≥90	8 (10.53)	9 (25.00)	17 (15.18)	
PI region				
High income	60 (78.95)	32 (88.89)	92 (82.14)	0.200
Nonhigh income	16 (21.05)	4 (11.11)	20 (17.86)	
PI region				
North America	27 (35.53)	14 (38.89)	41 (36.61)	0.942
Europe	20 (26.32)	9 (25.00)	29 (25.89)	
Other	29 (38.16)	13 (36.11)	42 (37.50)	

* *P* < 0.05

**Table 12:** Adjusted Logistic Regression Analysis of Association of Key Study Characteristics with Reference in Guidelines

Characteristic	Univariate analysis		Multivariate analysis	
	OR (95% CI)	*P*-value	OR (95% CI)	*P*-value
Time of registration				
2003–2013.06	1			
2013.06–2023	1.007 (0.415 ~ 2.443)	0.987		
Intervention				
Nonpharmacological related	1			
Pharmacological related	1.782 (0.767 ~ 4.137)	0.179		
Blinding				
None or open label	1			
Single	1.528 (0.385 ~ 6.070)	0.547		
Double or more	0.726 (0.301 ~ 1.752)	0.476		
Funder type				
None or departmental	1			
Industry or other external	1.456 (0.657 ~ 3.229)	0.355		
Recruitment				
Single center	1			
Multicenter	2.763 (1.143 ~ 6.683)	0.024*		
No. of participants				
<350	1			
≥350	1.667 (0.721 ~ 3.852)	0.232		
PI region				
Nonhigh SDI	1			
High SDI	2.857 (0.897 ~ 9.096)	0.076		
PI region				
<90	1			
≥90	2.833 (0.990 ~ 8.109)	0.052		
PI region				
High income	1			
Nonhigh income	0.469 (0.145 ~ 1.520)	0.207		
PI region				
North America	1			
Europe	–	0.785		
Other	0.865 (0.345 ~ 2.167)	0.756		

* *P* < 0.05

**Table 13:** Characteristics of Randomized Clinical Trials by Reuse of Prospective Data

Characteristic	Absence of reuse of prospective data, No. (%)	Presence of reuse of prospective data, No. (%)	Total, No. (%)	P
Time of registration				
2003–2013.06	66 (70.97)	15 (62.50)	81 (69.23)	0.423
2013.06–2023	27 (29.03)	9 (37.50)	36 (30.77)	
Primary purpose				
Treatment	80 (86.02)	19 (79.17)	99 (84.62)	0.474
Other	12 (12.90)	5 (20.83)	17 (14.53)	
Prevent	1 (1.08)	0 (0.00)	1 (0.85)	
Intervention				
Pharmacological	66 (70.97)	16 (66.67)	82 (70.09)	0.682
Nonpharmacological	27 (29.03)	8 (33.33)	35 (29.91)	
Intervention model				
Crossover	12 (12.90)	7 (29.17)	19 (16.24)	0.128
Parallel	80 (86.02)	17 (70.83)	97 (82.91)	
Factorial	1 (1.08)	0 (0.00)	1 (0.85)	
Arm				
2	74 (79.57)	20 (83.33)	94 (80.34)	0.795
3	14 (15.05)	4 (16.67)	18 (15.38)	
≥4	5 (5.38)	0 (0.00)	5 (4.27)	
Blinding				
None or open label	25 (26.88)	9 (37.50)	34 (29.06)	0.665
Single	10 (10.75)	2 (8.33)	12 (10.26)	
Double or more	58 (62.37)	13 (54.17)	71 (60.68)	
Funder type				
None or departmental	47 (50.54)	15 (62.50)	62 (52.99)	0.295
Industry or other external	46 (49.46)	9 (37.50)	55 (47.01)	
Recruitment				
Single center	35 (37.63)	11 (45.83)	46 (39.32)	0.538
Multicenter	55 (59.14)	13 (54.17)	68 (58.12)	
Missing	3 (3.23)	0 (0.00)	3 (2.56)	
No. of participants				
<350	65 (69.89)	16 (66.67)	81 (69.23)	0.760
≥350	28 (30.11)	8 (33.33)	36 (30.77)	
PI region				
Nonhigh SDI	22 (23.66)	3 (12.50)	25 (21.37)	0.235
High SDI	71 (76.34)	21 (87.50)	92 (78.63)	
PI region				
<90	79 (84.95)	21 (87.50)	100 (85.47)	1.000
≥90	14 (15.05)	3 (12.50)	17(14.53)	
PI region				
High income	75 (80.65)	21 (87.50)	96 (82.05)	0.560
Nonhigh income	18 (19.35)	3 (12.50)	21 (17.95)	
PI region				
North America	32 (34.41)	10 (41.67)	42 (35.90)	0.521
Europe	22 (23.66)	7 (29.17)	29 (24.79)	
Other	39 (41.94)	7 (29.17)	46 (39.32)	

* *P* < 0.05

## Discussion

This cross-sectional study provided the first analysis of the characteristics of 182 RCTs on GERD and esophageal HH conducted over the past 20 years, revealing substantial research wastage, with 86.7% of RCTs exhibiting at least one characteristic of study wastage. In total, we identified 129 RCTs (71.7%) in the scientific literature. Of the 117 RCTs available for full review, 90 (76.9%) had avoidable design defects. A total of 62 RCTs (53.0%) were judged to be adequately reported. In addition, five RCTs published in the most recent year (2023) were excluded; 36 trials (32.1%) were observed to be cited in the corresponding guidelines, and a total of 24 RCTs (20.5%) had prospective data reuse. In further analysis, double-blind and multi-blind designs and trials conducted in areas with an HAQ index of ≥90 was associated with lower odds of research waste.

The most effective way to minimize bias when evaluating new therapies in healthcare is through RCT studies^[32–36]^. The extent of the disease burden determines which RCTs should be conducted to improve the condition. However, previous studies^[37,38]^ have reported mismatches between disease burden and research funding. A systematic review of relevant previous studies and available evidence is essential for further research^[38–43]^. If such questions can be addressed satisfactorily with existing evidence, then no new research is required. GERD and esophageal HH are common conditions with diverse presentations. The symptoms of GERD overlap with those of other syndromes, which may influence pharmacological and surgical treatments^[44–47]^. In addition, GERD and HHs are treated by clinicians in many specialties, including general practitioners, internists, gastroenterologists, surgeons, emergency physicians, hospitalists, otolaryngologists, pulmonologists, obstetricians, and pediatricians. This has led to a wide range of perspectives^[48,49]^. Considering the diversity and complexity of GERD treatment, additional randomized controlled trials are needed to provide evidence-based medicine, but care should be taken to avoid research waste.

“Procedure” and “other” types of RCT may be underutilized in Asia. Procedure-type RCTs often require advanced medical equipment and technical support, which may limit their use across the continent. In a few Asian countries, patients and physicians may prefer more conservative treatments over surgery or other forms of treatment, which affects the development of procedure-based RCTs. The drug market in Europe is heavily regulated^[50–55]^, which may increase the difficulty of approval and time to initiate drug-type RCTs, and as such, drug-type RCTs are relatively underdeveloped on the European continent. Incidences of GERD and esophageal HH are relatively low in Oceania and South America. These regions may focus their research resources on other diseases or areas and discontinue exploration of interventions other than “drug” and “procedure” RCTs, resulting in insufficient “other*” types of RCTs.

The analysis of research waste in RCTs on GERD and hiatal hernia revealed that 86.7% of the trials exhibited at least one characteristic indicative of research waste. Investigators should be encouraged to conduct appropriately sized RCTs to ensure that the sample size is sufficient to detect the default effect size while using a higher level of blind design, such as double- or multiple-blindness. Strengthening the research capacity in geographic regions with low HAQ indices and providing methodological training, technical support, and financial assistance to help raise the standards of research design and execution in these areas can reduce research waste. Promoting international cooperation and sharing research experiences and resources, optimizing publication and reporting standards, and advocating for the development of transparent and comprehensive reporting guidelines such as the CONSORT statement can improve RCT quality on a global scale. Implementing these measures will help to ensure the accuracy and reproducibility of research results and reduce research waste.

Here, regions where RCTs were conducted were classified based on HAQ indices, and the concept of large RCTs was defined. In addition, we quantified the implementation of RCTs for different intervention types and identified limitations in the use of certain types of RCTs on several continents. Quantifying RCT performance is a complex task that is by no means limited to the five dimensions covered in this study; for example, multicenter clinical trials have additional intricacies in their design and execution that may affect the quality of the study, but these are not specifically differentiated or considered in our study. In addition, the analysis primarily relied on the ClinicalTrials.gov database, which contains information solely about RCTs that have been uploaded to the site. The assessment of reporting adequacy and risk of bias was based on the judgment of individual investigators, which may result in inconsistencies stemming from differences in the assessor’s experience, preferences, or understanding of the articles, affecting the reliability of the quantitative results. Although attempts have been made to minimize the impact of the quality of care on the universal ratio by adjusting HAQ values, the complex interactions of local differences in healthcare resources, policy environment, and research culture on the conduct and quality of RCTs may not be fully reflected in practical applications, which can lead to an incomplete assessment of global RCT conduct. However, the study aims to provide a preliminary overview for researchers and medical practitioners.

In an analysis of research waste^[25]^, first, quantifying research waste can be a challenging and difficult task. Waste was not limited to the three elements defined in this study. For example, repeated RCTs exploring low-priority issues with clear medical evidence may result in research wastage. Second, various endpoints were collected manually, which may have been related to measurement errors. However, we minimized measurement error by having two investigators evaluate each RCT independently, with disagreements resolved through discussion. Third, although ClinicalTrials.gov is a comprehensive registry of clinical trials, representing over 80% of all clinical studies listed on the World Health Organization (WHO) web portal^[56]^, the WHO also recognizes registries from other countries and regions^[57]^. However, RCTs from these registries were not included in the analysis. Fourth, delving into the potential reasons why or the underlying mechanisms by which small sample sizes or lack of external financial support are associated with research waste was beyond the scope of this study; however, this may be related to difficulties in obtaining effective infrastructure support for designing RCTs that have been linked with research waste.

## Conclusion

In this study, the GERD/HH-RCT research waste was significant, and there was a relative under-conducting of RCTs of different interventions in some continents. These findings suggest that there is room for improvement in study design, study implementation, publication of results, reuse of prospective data, and allocation of resources for the conduct of RCTs. This study may also provide evidence for the future conduct of medical RCTs to improve experimental design and reduce research waste.

## Data Availability

All data generated or analyzed during this study are included in this published article. The datasets used and/or analysed during the current study are available from the corresponding author on reasonable request.

## Assistance with the study

We thank who have devoted a lot to this study, including nurses, pathologists, further-study doctors, statisticians, reviewers, and editors. Thanks to Dr. Zhi-Hong Huang, Public Technology Service Center, Fujian Medical University. Feng-Qiong Liu, Experimental Center of School of Public Health, Fujian Medical University.
